# Land Subsidence in a Coal Mining Area Reduced Soil Fertility and Led to Soil Degradation in Arid and Semi-Arid Regions

**DOI:** 10.3390/ijerph16203929

**Published:** 2019-10-16

**Authors:** Kang Ma, Yuxiu Zhang, Mengying Ruan, Jing Guo, Tuanyao Chai

**Affiliations:** 1 School of Chemical and Environmental Engineering, China University of Mining & Technology (Beijing), Beijing 100083, China; mk@student.cumtb.edu.cn (K.M.); rmy@student.cumtb.edu.cn (M.R.); Guojing@student.cumtb.edu.cn (J.G.); 2 College of Life Science, University of Chinese Academy of Sciences, Beijing 100049, China

**Keywords:** Coal mining, land subsidence, soil nutrients, enzyme activities, microbial biomass

## Abstract

Underground coal mining in western China causes heavy land subsidence and alters the soil ecology. However, the effects of land subsidence on soil fertility are not currently known, and the key factors governing its impact remain unclear in sandy land. We investigated the effects of land subsidence induced by underground mining on the soil quality in western China. Soil samples were collected at 0–15 cm and 15–30 cm from control and subsidence areas in three coal mines. The results showed that the soil water content (SWC), clay and silt percentage, total nitrogen (TN), dissolved organic carbon (DOC), ammonia nitrogen (NH_4_^+^-N), nitrate nitrogen (NO_3_^-^-N), available phosphorus (AP), and available potassium (AK) of the subsidence areas were significantly lower than those of the control areas. The saccharase, urease, and alkaline phosphatase activities in the subsidence areas decreased compared to those in the control areas, while the sand percentage of soil tended to increase. Soil nutrient contents, bacterial quantities, and activities of soil enzymes were positively correlated with SWC. Redundancy analysis (RDA) showed that the soil particle size distribution, SWC, and electrical conductivity (EC) were the major environmental factors driving changes in soil properties. These results indicated that land subsidence induced by coal mining caused losses in surface soil water and nutrients, and ultimately led to soil quality degradation. Therefore, the reclamation of mining subsidence land might be necessary, especially in arid and semi-arid areas.

## 1. Introduction

Rapid economic development has stimulated the energy demand and further led to large-scale and high-intensity coal exploitation in China. However, underground mineral extraction has left large mining goafs, and these empty spaces might lead to sudden rock mass collapses or overburden cracking. Falling and collapsing disasters caused by goafs could result in surface collapse, ground fissures, and land subsidence, and ultimately trigger a series of serious chain reactions [1,2,3]. Land subsidence can directly affect the soil water and texture due to surface deformation, and cause an extensive decrease in the groundwater level [4,5]. In this sense, land subsidence triggered by underground mining can have an immediate and long-lasting impact on the soil quality, and thus on the ecosystem services [6,7]. 

Land subsidence causes substantial ecological and environmental problems, such as water and soil erosion, aggravation of land degradation, and declines in soil quality [8,9,10]. The soil structure and soil properties are both disturbed by land subsidence, which increases the average internal fraction angle value and reduces the average values of water and organic matter in soil [11]. In addition, land subsidence alters soil hydraulic properties, increases soil micropores, and decreases the field capacity [12]. Mining subsidence increases the soil infiltration depth and evaporation, while reducing the water-holding capacity of the upper soil layers. The loss of soil water inevitably leads to severe ecological deterioration. Furthermore, the plant density, coverage, and biomass are affected by land subsidence after mining [13]. Therefore, the ecosystem is disturbed by land subsidence and the soil quality further changes.

Soil physical and chemical properties play a central role in soil quality and are extensively used as soil quality indicators in the assessment and monitoring of degraded ecosystems [14]. Organic matter, nitrogen, and potassium contents are lower in mining subsided soils than in unmined soils, but the bulk density in mining subsided soils is higher than that in unmined areas [15]. The soil organic matter and available nutrients increase and the soil quality improves after the reclamation of a mining subsidence area [16]. Furthermore, soil enzymes and microbial biomass are closely related to soil physiochemical properties; act as driving forces in nutrient transformation; and play an important role in the cycling of soil nutrients such as carbon, nitrogen, and phosphorus [17,18,19]. Soil enzymes are released by plants, animals, and microorganisms and degrade complex organic molecules into absorbable simple molecules [20]. Therefore, change in soil nutrients, enzyme activities, and microorganisms can be used as sensitive indicators to understand soil quality variations due to coal mining.

With the increasing severity of mining subsidence worldwide, the scientific community is becoming increasingly interested in studying its impact., such as surface cracks, sand inrush disasters, soil hydraulic properties, and geological risks [2,6,21,22]. However, the responses of soil nutrients, enzymes, and microbes to mining subsidence remain poorly studied, particularly within fragile ecological regions of western China. Most subsided lands are located in western China and the subsidence areas affected by coal mining are continually increasing annually [23]. Land subsidence has altered the soil microbial community and decreased the soil microbial richness and diversity in the aeolian sand area of western China [24]. However, the impact of coal mining on soil quality in the arid and semi-arid region of western China has not been reported, and the effects of subsidence on the sandy soil properties are not clear. Therefore, this research is aimed at (1) evaluating the effects of the mining subsidence on the soil nutrients (C, N, P, and K), enzymes, and bacteria counts; (2) determining the key factors influencing the soil properties within mined and unmined soils in arid and semi-arid regions; and (3) examining the linkage between soil microorganisms and soil physicochemical properties after long-term land subsidence. Specifically, based on the high severity of the post-mining subsidence, we hypothesize that the soil nutrients and enzyme activities, as well as the abundance of bacteria, will decrease in soils of subsidence areas, compared to adjacent unmined areas. 

## 2. Materials and Methods

### 2.1. Study Area and Soil Sampling

**Figure 1:**
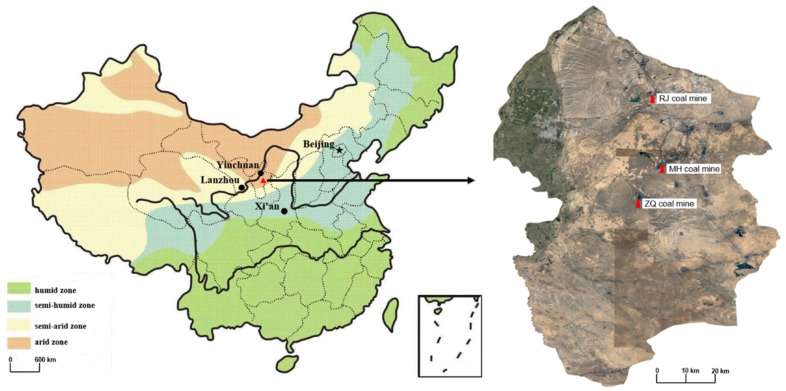
Location of the study area.

The study area is located in east-central Ningxia Province, Northwest China (106.36°E–106.74°E, 37.52°N–38.29°N) and the northwest margin of the Mu Us Desert (Figure 1). The climate of this region is arid or semi-arid, and this region is characterized by high evaporation and semifixed dunes, which form a fragile ecosystem. The mean annual temperature of this region is 6.7–8.8 °C, the mean annual precipitation is 255.2 mm, the mean annual evaporation is 2088.2 mm, the mean annual wind speed is 2.6 m·s^−1^, and the main monsoon season is July to October. The landform of the study area is mainly composed of a low mound platform, and the quaternary strata are widely distributed. The soil of the study areas consists of degraded sandy sierozem soil and can be classified as an Aridisol of sandy origin based on the Food and Agriculture Organization of the United Nations (FAO) soil classification system [25,26]. It is thus vulnerable to wind erosion and aeolian soil formation and characterized by a coarse texture and loose structure. The percent of vegetation cover is 10–30%, and the primary vegetation type is desert steppe, with plants such as *Caragana korshinskii* and *Artemisia ordosica*.

Six sampling areas were studied in three coal mines as showed in Table 1: subsidence areas and adjacent control areas of the RJ coal mine (RS and RC), MH coal mine (MS and MC) and ZQ coal mine (ZS and ZC). Surface soil samples (0–30 cm) with two layers were collected in October 2018. The land subsidence areas of RJ, MH, and ZQ were mined for five, three, and one year, respectively. The average depth of the coal seam was approximately 250 m, and the thickness of the coal seam was approximately 3–8 m in the study area. At each coal mine, the distance between the subsidence plot and control plot was approximately 300 m. In each coal mine, the subsidence and control regions had the same soil type and texture. 

The sampling followed a split plot experimental format with six different whole plots (RC, RS, MC, MS, ZC, and ZS). In each plot, three quadrants (5 × 5 m per quadrat) were established as three soil cores. In each quadrant, five samples were randomly collected at different soil depths (0–15 cm and 15–30 cm). Each sample (approximately 1 kg) of each category (RC15, RC30, RS15, RS30, MC15, MC30, MS15, MS30, ZC15, ZC30, ZS15, and ZS30) consisted of a mixture of 15 sub-samples at the same soil depth collected at 15 locations. All samples were collected by using a soil corer (10 × 12 cm in diameter and height, respectively) and transported to the laboratory; plant residues, stones, and other debris were carefully removed; and each sample was divided into two portions. One portion was air-dried and sieved to measure the soil properties, and the other portion was stored in a cooler at 4 °C to assay the saccharase, urease, and alkaline phosphatase activities and bacterial quantity.

### 2.2. Analysis of the Physical Properties of Soil Samples

The air-dried soil was passed through a 1 mm sieve, and the particle fractions were then determined in duplicate using a Mastersizer 2000 (Malvern Instruments, England). The soil particle size distribution was described with clay (0–2 µm), silt (2–50 µm), fine sand (50–250 µm), moderate sand (250–500 µm), and coarse sand (500–1000 µm) following the taxonomy of the United States Department of Agriculture (USDA)/FAO. Soil water contents (SWC) were determined gravimetrically from the mass loss following oven-drying at 105 °C to a constant weight. The pH was measured with a 1:2.5 m:v soil-to-water suspension by a glass electrode. Soil electrical conductivity (EC) was measured with a 1:5 m:v soil-to-water suspension at 25 ℃ after shaking for 30 minutes by a microprocessor conductivity meter (Shanghai Youke Instrument Factory, Shanghai, China) [27].

Soil organic carbon (SOC) was measured using the colorimetric method after digestion with K_2_Cr_2_O_7_/H_2_SO_4_ at 165 °C in an oil bath [28]. Water-extractable dissolved organic carbon (DOC) was extracted with distilled water (1:5 v:v) and measured with a TOC (total dissolved organic) analyzer (Liqui TOC II, Elementar, Germany) [29]. The semimicro Kjeldahl method was used for the determination of total nitrogen (TN). The ammonia nitrogen (NH_4_^+^-N) and nitrate nitrogen (NO_3_^−^-N) were extracted with 1 M KCl (1:1 v:v) and measured by a continuous-flow autoanalyzer [30]. Total P (TP) was determined by molybdate colorimetry after digestion with a mixed reagent of H_2_SO_4_ and HClO_4_ at 250 °C. Available phosphorus (AP) and available potassium (AK) were extracted and measured using the methods described by Bao [31]. 

Soil enzyme activities were measured as previously described [32,33]. Saccharase (EC 3.2.1.26) activity was measured as follows: 0.2 mL of toluene, 5 mL of phosphate buffer (pH 5.5), and 15 mL of 8% sucrose solution were added to 5 g of sample. After incubating the samples for 24 h at 37 °C and the addition of 0.5 mL of 3,5-dinitrosalicylic acid, the formation of 3-amino-5-nitrosalicylic acid was determined spectrophotometrically at 508 nm. Urease (EC 3.5.1.5) activity was routinely measured as follows: 1 mL of 10% urea substrate solution, 0.25 mL of toluene, and 0.75 mL of citrate buffer (pH 6.7) were added to 1 g of sample. After incubating the samples for 24 h at 37 °C, the formation of ammonium was determined spectrophotometrically at 578 nm. The determination of alkaline phosphatase (EC 3.1.3.1) was conducted using p-nitrophenyl phosphate (in modified universal buffer at pH 11.0) as a substrate; after incubation at 37 °C for 1 h, the liberated phenol was measured at 400 nm using a spectrophotometer.

The colony-forming units (CFU) of bacteria were determined by the serial dilution method. Bacterial populations were counted following the standard dilution plate technique. Ten grams of sample was taken and dissolved in sterile water up to a volume of 90 mL, which was further serially diluted to obtain 10^−4^ dilution. From these diluted samples, 1 mL of solution was dispensed for three replicates, and media for the growth of microorganisms were then added. The populations of microorganisms were determined from the number of microbes multiplied by the dilution factor for each sample.

### 2.3. Data Analysis

All analytical determinations were performed in triplicate, and SPSS 23.0 (IBM, Armonk, NY, USA) was applied for a one-way analysis of variance (ANOVA Company city country) at the 0.05 significance level. Pearson correlations were used to assess relationships between different soil variables (physicochemical properties and biological activities of the soil). Principal component analysis (PCA) and redundancy analysis (RDA) were performed to determine the overall differentiation of the soil indicators among the samples. For PCA, soil physical, chemical, and biochemical properties were utilized, and the tested areas were agglomerated into groups with differing measured soil parameters. PCA and RDA were conducted using the CANOCO 5.1 (Microcomputer Power, Ithaca, NY, USA) software package.

## 3. Results

### 3.1. Effect of Land Subsidence on Soil pH, SWC, EC, and Texture

**Figure 2:**
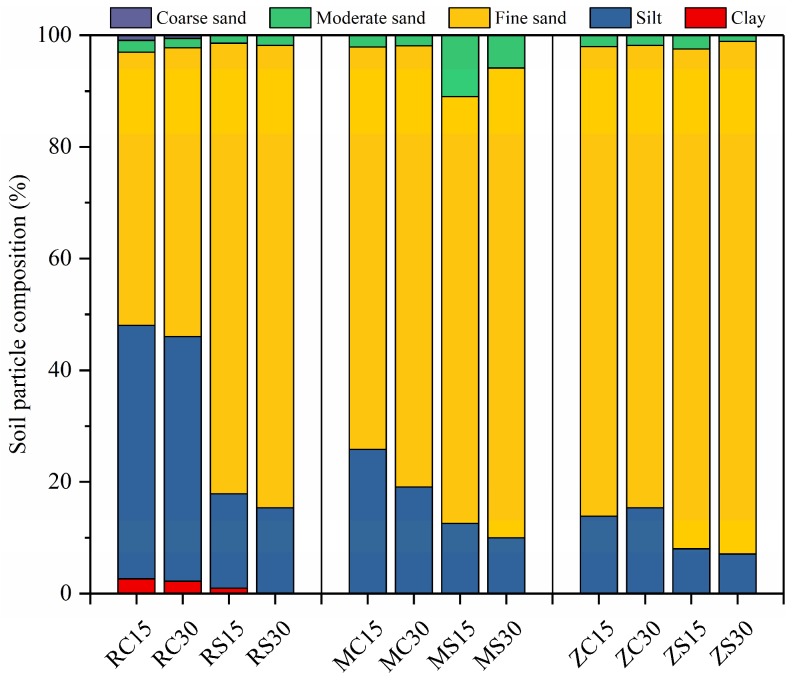
Soil particle size distribution of samples. Soil particle classifications: Clay (0–5 µm); silt (5–50 µm); fine sand (50–250 µm); moderate sand (250–500 µm); and coarse sand (500–1000 µm).

The soil particle size distribution is indicated in Figure 2. As shown, the dominant soil particle composition in the RJ coal mine consisted of sand and silt, while sand was the major component of the samples taken from the MH and ZQ coal mines. As shown in Figure 2, differences in clay contents, silt contents, and sand contents were found among the subsidence areas and control areas. The clay and silt contents decreased in subsidence areas compared to those in control areas, and sand contents increased. These results showed that the particle size distribution of the three coal mines was mainly composed of sand, and land subsidence has resulted in an increase in sand content and a decrease in clay and silt contents. The very serious water and wind erosion in this area is one of the most important contributors to soil desertification, and land subsidence has exacerbated the issues.

The soil pH, SWC, and EC of the samples demonstrated different trends, as shown in Table 1. The soil pH of all studied areas ranged from 8.01 to 8.13 and did not differ among samples collected from the control and subsidence areas. SWC ranged from 3.18% to 9.17% in the three coal mines, and the average SWC was 8.22% in RJ, 7.40% in MH, and 4.89% in ZQ. The soil EC ranged from 34.85 to 66.20 μS·cm^−1^, and the average values were 57.23, 47.94, and 35.63 μS·cm^−1^ in the RJ, MH, and ZQ coal mines, respectively. Comparing the subsidence and control areas in the same coal mine, the soil pH did not differ, whereas the SWC declined from the control to subsidence area, with an average decrease of approximately 15.67% in RJ, 10.80% in MH, and 32.15% in ZQ. The soil EC increased in subsidence areas compared to that in control areas by approximately 7.15% in RJ, 12.64% in MH, and 9.36% in ZQ. These results showed that the soil water content of the three coal mines was deficient; SWC decreased and soil EC increased. The reason for this difference could be the water loss caused by the higher evaporation and infiltration rates induced by land subsidence.

### 3.2. Effect of Land Subsidence on Soil Nutrients

**Figure 3:**
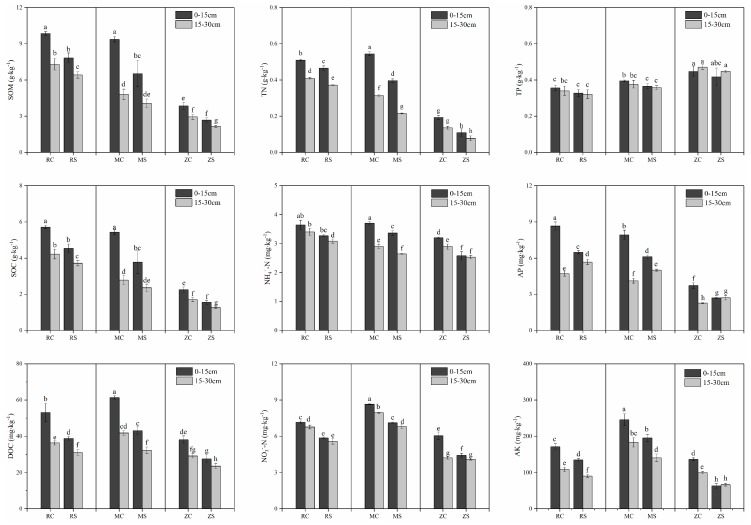
Changes in soil nutrients in the control and subsidence areas of three coal mines. Different lowercase letters indicate significant differences at 0.05 (*p* < 0.05) levels. SOM: soil organic matter; SOC: soil organic carbon; DOC: dissolved organic carbon; TN: total nitrogen; TP: total phosphorus; AP: available phosphorus; AK: available potassium.

The soil nutrients of the three coal mines were observed and are shown in Figure 3. In the current study, SOC and DOC ranged from 1.25 to 5.72 g·kg^−1^ and 23.61 to 61.41 mg·kg^−1^, with average contents of 3.28 and 43.28 mg·kg^−1^, respectively, in the three coal mines. TN ranged from 0.08 to 0.58 g·kg^−1^ in the three coal mines, with an average content of 0.29 g·kg^−1^. The NH_4_^+^-N and NO_3_^−^-N contents ranged from 2.54 to 3.65 mg·kg^−1^ and 4.12 to 10.65 mg·kg^−1^, with an average content of 6.31 and 3.10 mg·kg^−1^, respectively. TP and AP varied from 0.32 to 0.45 g·kg^−^1 and 2.28 to 7.93 mg·kg^−1^ in the three coal mines, with average contents of 0.29 and 5.02 g·kg^−1^, respectively. In addition, AK ranged from 63.63 to 265.86 g·kg^−1^ in the three coal mines, with an average content of 138.33 g·kg^−1^.

Compared to their levels in control areas, soil nutrients in the subsidence areas appeared to be decreased. The SOC and DOC contents of subsidence areas compared to those in control areas decreased by 16.25–28.51% and by 20.53–26.06%, respectively. Moreover, the TN, NH_4_^+^-N, and NO_3_^−^-N contents of the subsidence areas decreased by 9.88–43.15%, 7.99–15.89%, and 14.42–32.21%, respectively (*p* < 0.05 for all above nutrients). The soil TP contents varied slightly, and there was no significant difference in the TP of control and subsidence areas within individual mines. The soil AP content in the topsoil (0–15 cm) of the control areas was significantly higher than that in the subsidence areas by 22.84–27.57%, while the concentration at 15–30 cm was 19.10–20.43% lower in the control areas than in the subsidence areas (*p* < 0.05). The tendency of soil AK was similar to that of soil carbon and nitrogen, and the soil AK decreased by 18.89–43.47% in the subsidence areas compared to that in the control areas (*p* < 0.01). All these results showed that the contents of soil nutrients in subsidence areas were lower than those in control areas. Multiple reasons, such as differences in the geological structure of different zones and the subsidence occurrence time, resulted in these variations. Additionally, the nutrient contents decreased with an increasing soil depth, and the rates of decrease differed between the control and subsidence areas. 

With soil depth as a factor, the SOC of the control areas decreased from 0–15 cm to 15–30 cm by 26.00% in RJ, 48.64% in MH, and 24.15% in ZQ, while these percent decreases were 18.01%, 37.68%, and 19.38%, respectively, in the subsidence areas. The subsidence areas had lower decrease rates over soil depth than control areas, and similar trends were also found for DOC, TN, NH_4_^+^-N, NO_3_^−^-N, AP, and AK. These results showed that land subsidence significantly altered the vertical distribution of soil nutrients, and the effects might result from vertical leakage from the top layer to the deeper layer in the subsidence areas. 

Underground collapse has an effect on the surface soil and results in the loss of soil nutrients, yet the degree of this loss among the studied coal mines was different. The plots of the RJ and MH coal mines had been exploited more than three years, while that of the ZQ coal mine had been exploited for one year. The degree of soil nutrient loss in the ZQ coal mine was more drastic than that in the RJ and MH mines, including the SOC, TN, NH_4_^+^-N, AP, and AK contents. This result implied that the effect of land subsidence on soil nutrients might be related to time and that the soil nutrients tended to recover by self-healing after three years of land subsidence. 

Furthermore, the background contents of SOC, TN, TP, AP, and AK in the study area were 0.55–8.07, 0.02–0.72, 0.35–0.7, 4.4–11.3, and 20–300 mg·kg^−1^, respectively. The three coal mines were in the sandy land of western China, and the soil particles were mainly composed of medium and fine sand. Soil nutrients in coal mines were deficient, demonstrating that the soils of the current study areas were infertile and susceptible to disturbances.

### 3.3. Effect of Land Subsidence on Soil Enzymes and Bacterial Quantity

**Figure 4:**
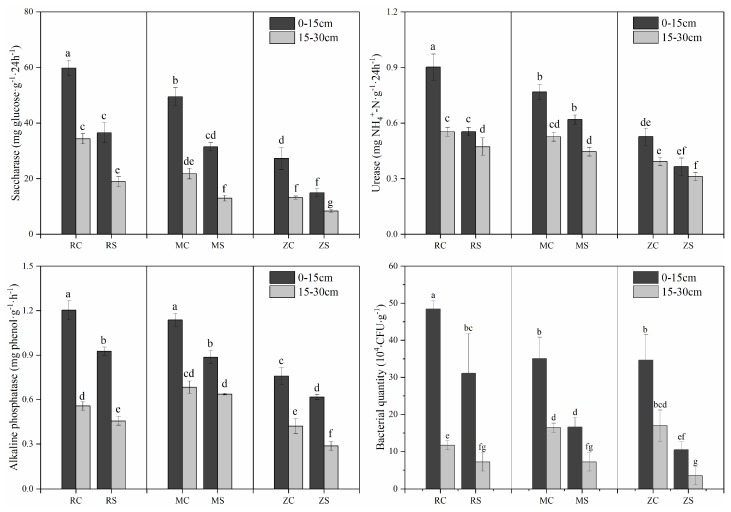
Changes in soil enzyme (saccharase, urease, and alkaline phosphatase) activities and bacterial quantity in the control and subsidence areas of three coal mines. Different lowercase letters indicate significant differences at 0.05 (*p* < 0.05) levels.

The activities of three enzymes (saccharase, urease, and alkaline phosphatase) in the RJ, MH, and ZQ coal mines are displayed in Figure 4. All soil enzyme activities decreased with an increasing depth in both the control and subsidence areas of the current study, but the response to land subsidence was not consistent. Lower activities of saccharase, urease, and alkaline phosphatase were found in subsidence areas than in control areas (*p* < 0.01 for all these enzymes). In contrast to the control areas, in the subsidence areas, soil saccharase decreased by an average of 42.15%, 37.66%, and 42.25% in the RJ, MH, and ZQ coal mines, respectively. Similarly, the urease and alkaline phosphatase activities of the subsidence areas were lower than those of the control areas; these decreases were 29.49% in RJ, 17.74% in MH, and 26.37% in ZQ for urease and 20.34% in RJ, 16.44% in MH, and 14.86% in ZQ for alkaline phosphatase. Therefore, all results implied that the soil biological enzymes of coal mines were influenced by land subsidence, which might be due to the disturbance of environmental factors and soil nutrient variations. The number of soil bacteria was observed and is shown in Figure 4. The soil microbial counts were lower in soil samples from mining subsidence areas than in soil from control areas. Compared with that in non-subsidence soil samples, the number of bacteria decreased, suggesting that land subsidence had a significant effect on soil microbial abundance. An analysis of different coal mines indicated that the soil fertility quality in coal mines was deficient, especially in ZQ coal mines. Higher microbial population levels were counted in topsoils, which might be due to the abundant soil water at 0–15 cm. The data also showed that microbial numbers were larger, but not significantly larger, after three years of land subsidence. Therefore, the bacterial count explained the results more intuitively and further emphasized that the soil quality decreased after land subsidence and tended to recover.

### 3.4. Relationships among Soil Physiochemical Properties, Nutrients, and Enzyme Activities

**Figure 5:**
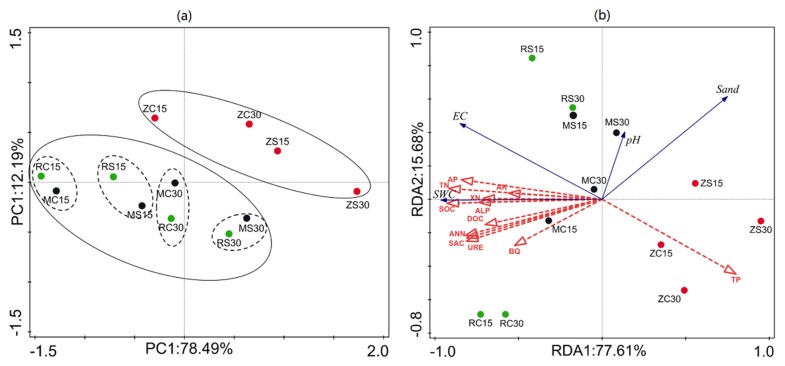
(**a**) Principal component analysis (PCA) of soil physical chemical and biological properties; (**b**) ordination triplot (RDA) of environmental variables, soil nutrients, and enzyme activities. SOC: soil organic carbon; DOC: dissolved organic carbon; TN: total nitrogen; TP: total phosphorus; AP: available phosphorus; AK: available potassium; SAC: saccharase; URE: urease; ALP: alkaline phosphatase; BQ: bacterial quantity.

Principal component analysis (PCA) was applied to the results in our study, as shown in Figure 5. The first two component axes explained 78.49% and 12.19% of the total variance of soil properties. Samples for the soil properties were roughly grouped, where the distance between samples represented the extent of differences. Groups MC15 and MC30 were close to groups RC15 and RC30, implying that the soil properties of the MH and RJ coal mines were similar. In addition, the distances between samples from subsidence areas and control areas in MH and RJ were shorter than those in ZQ, indicating that the effects of land subsidence on soil properties in the ZQ coal mines were more severe than those in the other coal mines. Therefore, the clusters in Figure 5 explained the results obtained before and further emphasized that soil quality is related to time, as soil quality tended to recover by self-healing after some years of land subsidence.

**Figure 6:**
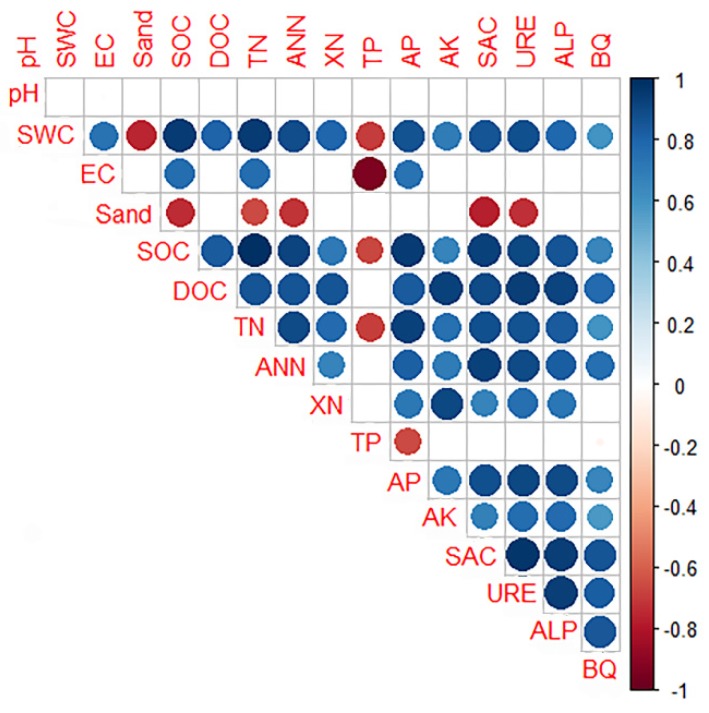
Correlations of soil physicochemical characteristics, nutrients, and enzyme activities. SWC: soil water content; EC: electrical conductivity; SOC: soil organic carbon; DOC: dissolved organic carbon; TN: total nitrogen; TP: total phosphorus; AP: available phosphorus; AK: available potassium; SAC: saccharase; URE: urease; ALP: alkaline phosphatase; BQ: bacterial quantity.

To further explore the relationships between physical, chemical, and biological properties, a redundancy analysis (RDA) triplot was constructed, as shown in Figure 5. RDA1 and RDA2 explained 77.61% and 15.68% of the variance, respectively. The sand percentage and SWC were the strongest determinants and exhibited a negative and positive relationship, respectively. In addition, the arrow lines of sand percentage and SWC were the longest, indicating that both of them were the major environmental factors driving the changes in soil nutrients and enzyme activities. The distribution patterns for samples of the control areas and subsidence areas indicated that land subsidence affected the soil nutrients and enzymes. Furthermore, the correlation between the physicochemical properties and enzyme activities for the soil was analyzed by Pearson’s correlation (Figure 6). 

Three enzyme activities and bacterial quantities were positively correlated with each other. Saccharase, urease, alkaline phosphatase, and bacterial quantity were significantly positively correlated with SWC, SOC, TN, DOC, NH_4_^+^-N, AP, and AK, but not with soil pH, EC, and TP. In addition, SWC was significantly correlated with most of the soil properties. The results implied that there were significant correlations among the soil physicochemical properties, nutrients, and enzymes. The RDA confirmed the results of the correlation analysis and demonstrated that the main environmental factors determining the soil nutrients and enzyme activities were SWC and soil EC.

## 4. Discussion

### 4.1. Land Subsidence Reduced SWC, and Increased EC and Sand Content

Land subsidence in coal mining can induce several types of ecological environmental destruction, such as land destruction, soil erosion, and vegetation degeneration, further altering the soil properties. Studies have shown that land subsidence significantly influences soil properties. Wang investigated coal mines in arid areas and demonstrated that the drying trend in the arid regions was due to underground mining [5]. Yang also proved that land subsidence reduced SWC and soil cohesion [11]. SWC is a key factor of soil influencing the soil quality, particularly in our study area, which is located in arid and semi-arid regions of China. 

Soil texture is measured using the percentages of clay, silt, and sand, and is critical for understanding the transportation and storage of soil water and nutrients, as well as the mineralization of organic matter content [34]. Land subsidence caused dense surface cracks and this may result in the loss of silt and clay, an increased sand percentage, and a tendency for soil to undergo desertification. Additionally, the SWC in the subsidence areas was significantly lower than that in the control areas in the current study. This reduction in SWC could be attributed to the dense surface cracks caused by coal seam exploitation destroying the soil structure and increasing the total soil porosity; thus, land subsidence increases the evaporation and infiltration capacity of soil [6,11,12]. Moreover, the higher soil EC observed in the subsidence areas than in the control areas might be due to the high evaporation of soil water and low rainfall. Salt in the soil can be transported and accumulate in the topsoil due to higher ion concentrations [24]. Soil salinity can affect the growth of plants and inhibit sensitive microbial populations. In addition, the migration, distribution, and use efficiency of water and nutrients were affected by soil texture; the soil particle size distribution can affect the soil water storage capacity, such that a cycle of progressive soil deterioration is established. Therefore, land subsidence can significantly increase soil water evaporation and infiltration and decrease the clay and silt contents of soil, leading to an SWC reduction and accelerating soil drought, salinization, and desertification, particularly in arid and semi-arid areas.

### 4.2. Soil Nutrients were Reduced by Leaching and Infiltration in Land Subsidence

The present study showed that the contents of SOM, SOC, DOC, TN, NH4+-N, NO_3_--N, AP, and AK in subsidence areas were significantly lower than those in control areas, indicating that the loss of soil nutrients was triggered by land subsidence. These findings might be explained by complex biochemical cycling processes, such as plant root function, microbial community variation, soil solute vertical leaching, and crack leakage [24,35]. 

Land subsidence altered the SWC and soil EC, resulting in plant and soil microbe disturbances. The nutrient cycle was affected by land subsidence and led to a lower soil nutrient content. In addition, different nutrient elements showed different variations. It was reported that with a considerable increase in plant coverage and plant biomass, the accumulation of SOC and TN could be improved directly or indirectly [36,37]. Li found that soil TN increased after the reclamation of soil in a subsidence area affected by mining activities. The disturbance of surface plants by land subsidence can inhibit the process of biological C and N fixation and ultimately change the C and N stocks in subsidence areas [38,39]. The contents of soil C and N were lower in the subsidence areas than in the control areas in the current study. A similar trend was also found for AP and AK. In addition, there was no significant difference in TP between the subsidence and control areas. It has been reported that phosphorus retention depends on particulate phosphorus deposition from the air, soil adsorption, and other sediments, while land subsidence has a limited effect [40]. 

All soil nutrient concentrations gradually decreased with soil depth in the current study. This finding was consistent with previous studies and might be explained by the better growth conditions in topsoil layers than in lower layers. Liu studied the soil profile after mining and found that the vertical distribution of SOM, TN, AP, and AK decreased with depth [41]. The distribution characteristics of SOM in subsidence areas of coal mining were researched, and the results showed that the SOM contents gradually decreased with an increasing depth of the soil profile [42]. Nevertheless, the vertical distributions of soil nutrients in subsidence and control areas were different in the current study; specifically, the vertical decreases in SOC DOC, TN, NH_4_^+^-N, NO_3_^−^-N, AP, and AK with depth in the control areas were higher than those in the subsidence areas. The results might be due to the leaching of soil nutrients through surface fractures [24]. Complicated processes of geology and mechanics occurred, inducing surface subsidence and cracks. The soil nutrients infiltrated from the topsoil layer to the deeper layers through surface fractures, which resulted in an increase in nutrients and vertical distribution variations. Certainly, the AP content in subsidence areas increased at 15–30 cm compared to that at 0–15 cm in the current study, further demonstrating the vertical leakage of soil nutrients in subsidence areas.

The loss of soil nutrients by land subsidence over one year (the ZQ coal mine) was more severe than that over five or three years (the RJ and MH coal mines). Soil properties can recover by self-healing, but this process is slow, and the soil had not recovered five years after land subsidence in the current study. The managers of coal mines could use engineering technology methods, such as crack backfill and soil reclamation techniques, to mitigate these effects. Therefore, artificial reclamation of mining subsidence land might be necessary and feasible if performed effectively [43]. 

Soil property variations can be triggered by intrinsic or extrinsic sources of variability. Soil intrinsic variability is natural variation, and extrinsic variability is caused by environmental factors [44]. Underground coal development induces land destruction, including a decreased soil water content (SWC), increased soil permeability, and intensified soil erosion and desertification, directly or indirectly influencing soil nutrient dynamic cycling and ultimately changing the soil properties of subsidence areas.

### 4.3. Reduction of Soil Microbes Led to Nutrient Content Reduction in Land Subsidence

Soil enzymes are mainly derived from plant root exudates, soil animals, and especially soil microbes [20,45]. Our study demonstrated that soil enzymatic activities and the abundance of CFUs of bacteria gradually decreased with depth in all areas of the current study, which is probably due to declines in soil moisture and temperature with depth. Better growth conditions in the topsoil layer than in deeper layers can promote the growth of plants and microorganisms, as demonstrated by many previous studies. In the current study, a comparison of the activity of enzymes (saccharase, urease, and alkaline phosphatase) in all plots revealed that land subsidence influences enzyme activities and that different enzymes respond to subsidence similarly. Saccharase, urease, and phosphatase participate in soil C, N, and P cycling and affect soil fertility conditions [46]. Saccharase can change cellulose, starch, and saccharides into lower-molecular-weight compounds [47]. Urease aides the hydrolysis of urea into carbon dioxide and ammonia. Phosphatase enzymes catalyze the processes of organic phosphorus mineralization and immobilization to affect the potential supply of organic phosphorus. These enzyme activities in subsidence areas were lower than those in control areas in the same coal mine, as well as the bacterial quantities. From the results presented here, we argue that soil nutrients tend to gradually get lost over time as a result of the decrease of water and microorganisms in the soil, both of which are key components for soil fertility after land subsidence. The soil erosion of subsidence areas can become worse over time as a consequence of soil nutrients loss and microbes reduction. Therefore, our research indicated that land subsidence greatly disturbed biological activities, thereby changing the activities of soil enzymes and ultimately reducing the contents of soil nutrients.

### 4.4. Land Subsidence Led to Soil Segradation and Desertification

Soil quality represents the capacity of soil to promote plant and animal productivity and maintain or enhance water and air quality. In this study, physical, chemical, and biological soil quality indicators were observed, and the soil quality of the control and subsidence areas was very different. The soil samples of subsidence areas exhibited lower soil nutrient contents and enzyme activities than the control soil samples. Soil enzymes are linked to soil nutrient dynamics, such as C cycling, N cycling, and P cycling, and have effects on chemical and biological processes in soil ecosystems. A previous study found that soil nutrients could promote increases in soil animals, plants, and microbial populations and that SOC and TN supply substrates promote enzyme synthesis, leading to changes in soil enzymes [20,48]. 

Soil nutrients and soil enzyme activities were closely correlated with each other and were affected by environmental factors in the current study. A previous study demonstrated that soil moisture is an important factor affecting enzyme activities. The improvement of SWC provided a favorable environment for plants and microorganisms, thereby enhancing the synthesis of soil enzymes, which was consistent with our results [17]. Moreover, evaporation is greater than rainfall in the sandy land of western China, resulting in salt accumulation in soils. Therefore, soil EC is also expected to be a stressful environmental factor affecting soil plants and microorganisms, further affecting soil nutrients and enzymes. In addition, the sand content manifests in the severe degradation of soil quality. Therefore, the soil particle distribution, SWC, and soil EC were found to be the main environmental factors affecting the sandy land of western China. Furthermore, decreased numbers of microorganisms indicated that land subsidence effectively induces soil quality degradation, and microbial populations can be used as important biological indicators to measure damaged ecosystems. The land subsidence induced by coal mining caused a loss of soil water and soil salinization, which led to a reduction in soil quality.

## 5. Conclusions

Our study showed that land subsidence had severe effects on soil properties, such as the SWC, soil EC, soil particle-size distribution, nutrients, enzyme activities, and bacterial quantity. Land subsidence caused ground cracks and the vertical leakage of nutrients. The soil nutrient content of the top layer in the subsidence areas was significantly lower than that in the control areas. Significant correlations were found between soil physicochemical and biological properties, suggesting that soil microorganisms were closely regulated by soil nutrient dynamics and had positive relationships with soil nutrients. Land subsidence changed environmental factors and disturbed the mineralization of C, N, and P cycling processes, which resulted in soil nutrient and enzyme variations. In addition, the soil particle distribution, SWC, and soil EC were determined to be the main environmental factors impacted by subsidence in the sandy land of western China. In short, these findings highlight the impacts of coal mining subsidence on soil properties and this work can greatly contribute to understanding soil conditions and the environmental risks to the local population.

## Figures and Tables

**Table 1:** Soil physicochemical characteristics of samples.

Samples	Depth (cm)	pH	SWC (%)	EC (µS·cm^−^^1^)
RC15	0–15	8.01 ± 0.02bc	9.17 ± 0.10a	55.06 ± 2.70bc
RC30	15–30	8.04 ± 0.02b	8.36 ± 0.01c	52.50 ± 0.64c
RS15	0–15	8.08 ± 0.06ab	7.72 ± 0.05d	66.20 ± 2.19a
RS30	15–30	8.13 ± 0.03a	7.08 ± 0.01f	55.15 ± 1.70b
MC15	0–15	8.02 ± 0.06ab	8.63 ± 0.06b	46.40 ± 1.63d
MC30	15–30	8.11 ± 0.02a	6.32 ± 0.16h	45.85 ± 1.98d
MS15	0–15	8.04 ± 0.02b	7.58 ± 0.08e	51.80 ± 1.48c
MS30	15–30	8.07 ± 0.04ab	6.98 ± 0.03g	47.40 ± 0.49d
ZC15	0–15	8.08 ± 0.01b	5.79 ± 0.01i	35.95 ± 0.99ef
ZC30	15–30	8.09 ± 0.03ab	5.41 ± 0.05j	32.10 ± 1.48f
ZS15	0–15	8.03 ± 0.03b	4.45 ± 0.03k	39.60 ± 3.75e
ZS30	15–30	8.04 ± 0.01b	3.18 ± 0.01k	34.85 ± 1.41ef

SWC: soil water content; EC: electrical conductivity; values are the means ± S.D; different lowercase letters indicate significant differences between samples at 0.05 (*p* < 0.05) levels.

## References

[1] Ju J., Xu J. (2015). Surface stepped subsidence related to top-coal caving longwall mining of extremely thick coal seam under shallow cover. Int J Rock Mech Min.

[2] Luan H., Lin H., Jiang Y., Wang Y., Liu J., Wang P. (2018). Risks induced by room mining goaf and their assessment: A case study in the Shenfu-Dongsheng mining area. Sustain..

[3] Saeidi A., Deck O., Verdel T. (2009). Development of building vulnerability functions in subsidence regions from empirical methods. Eng. Struct..

[4] Tripathi N., Singh R.S., Singh J.S. (2009). Impact of post-mining subsidence on nitrogen transformation in southern tropical dry deciduous forest, India. Environ. Res..

[5] Wang Y., Bian Z., Lei S., Zhang Y. (2017). Investigating spatial and temporal variations of soil moisture content in an arid mining area using an improved thermal inertia model. J. Arid. Land.

[6] Machowski R., Rzetala M.A., Rzetala M., Solarski M. (2016). Geomorphological and hydrological effects of subsidence and land use change in industrial and urban areas. Land Degrad. Dev..

[7] Sun Q., Zhang J., Zhang Q., Zhao X. (2017). Analysis and prevention of geo-environmental hazards with high-intensive coal mining: A case study in China’s western eco-environment frangible area. Energies.

[8] Chen Y., Zhang J., Zhou A., Yin B. (2018). Modeling and analysis of mining subsidence disaster chains based on stochastic Petri nets. Nat. Hazards.

[9] Shepley M.G., Pearson A.D., Smith G.D., Banton C.J. (2008). The impacts of coal mining subsidence on groundwater resources management of the East Midlands Permo-Triassic Sandstone aquifer, England. Q. J. Eng. Geol. Hydroge..

[10] Wu Q., Pang J., Qi S., Li Y., Han C., Liu T., Huang L. (2009). Impacts of coal mining subsidence on the surface landscape in Longkou city, Shandong Province of China. Environ. Earth Sci..

[11] Yang D., Bian Z., Lei S. (2016). Impact on soil physical qualities by the subsidence of coal mining: A case study in Western China. Environ. Earth Sci..

[12] Wang J., Wang P., Qin Q., Wang H. (2017). The effects of land subsidence and rehabilitation on soil hydraulic properties in a mining area in the Loess Plateau of China. Catena..

[13] Yang Y., Erskine P.D., Zhang S., Wang Y., Bian Z., Lei S. (2018). Effects of underground mining on vegetation and environmental patterns in a semi-arid watershed with implications for resilience management. Environ. Earth Sci..

[14] Mukhopadhyay S., Maiti S.K., Masto R.E. (2014). Development of mine soil quality index (MSQI) for evaluation of reclamation success: A chronosequence study. Ecol. Eng..

[15] Guo X., Zhao T., Chang W., Xiao C., He Y. (2018). Evaluating the effect of coal mining subsidence on the agricultural soil quality using principal component analysis. Chil. J. Agric. Res..

[16] Bi Y., Zhang Y. (2014). Role of the different planting age of seabuckthorn forests to soil amelioration in coal mining subsidence land. Int. J. Coal. Sci. Technol..

[17] Brockett B., Prescott C., Grayston S. (2012). Soil moisture is the major factor influencing microbial community structure and enzyme activities across seven biogeoclimatic zones in western Canada. Soil. Biol. Biochem..

[18] Qi R., Li J., Lin Z., Li Z., Li Y., Yang X., Zhang J., Zhao B. (2016). Temperature effects on soil organic carbon, soil labile organic carbon fractions, and soil enzyme activities under long-term fertilization regimes. Appl. Soil. Ecol..

[19] Zhang C., Liu G., Xue S., Wang G. (2016). Soil bacterial community dynamics reflect changes in plant community and soil properties during the secondary succession of abandoned farmland in the Loess Plateau. Soil. Biol. Biochem..

[20] Burns R.G., DeForest J.L., Marxsen J., Sinsabaugh R.L., Stromberger M.E., Wallenstein M.D., Weintraub M.N., Zoppini A. (2013). Soil enzymes in a changing environment: Current knowledge and future directions. Soil. Biol. Biochem..

[21] Li L., Wu K., Hu Z., Xu Y., Zhou D. (2017). Analysis of developmental features and causes of the ground cracks induced by oversized working face mining in an aeolian sand area. Environ. Earth. Sci..

[22] Yan W., Dai H., Chen J. (2018). Surface crack and sand inrush disaster induced by high-strength mining: Example from the Shendong coal field, China. Geosci. J..

[23] Wang J., Qin Q., Hu S., Wu K. (2016). A concrete material with waste coal gangue and fly ash used for farmland drainage in high groundwater level areas. J. Clean Prod..

[24] Shi P., Zhang Y., Hu Z., Ma K., Wang H., Chai T. (2017). The response of soil bacterial communities to mining subsidence in the west China aeolian sand area. Appl. Soil. Eco..

[25] International Soil Reference and Information Centre, AGL, International Union of Soil Sciences Working Group, FAO (2006). World reference base for soil resources. World Soil Resour. Rep..

[26] Shi X., Yu D., Sun W., Wang H., Zhao Q., Gong Z. (2004). Reference benchmarks relating to great groups of genetic soil classification of China with soil taxonomy. Chin. Sci. Bull..

[27] Gao Y., Wang J., Guo S., Hu Y., Li T., Mao R., Zeng D. (2015). Effects of salinization and crude oil contamination on soil bacterial community structure in the Yellow River Delta region, China. Appl. Soil. Eco..

[28] Bowman R.A., Cole C.V. (1978). An exploratory method for fractionation of organic phosphorus from grassland soils. Soil. Sci..

[29] Zhang X., Yang Y., Zhang C., Niu S., Yang H., Yu G., Wang H., Blagodatskaya E., Kuzyakov Y., Tian D. (2018). Contrasting responses of phosphatase kinetic parameters to nitrogen and phosphorus additions in forest soils. Func. Ecol..

[30] Page A.L., Miller R.H., Keeney D.R. (1982). Nitrogen-availability Indices: Methods of Soil Analysis, Part 2, Chemical and Microbiological Properties.

[31] Bao S. (2000). Soil and Agricultural Chemistry Analysis.

[32] Tabatabai M.A., Bremner J.M. (1969). Use of p-nitrophenyl phosphate for assay of soil phosphatase activity. Soil Biol. Biochem..

[33] Zhang C., Xue S., Liu G., Song Z. (2011). A comparison of soil qualities of different revegetation types in the Loess Plateau, China. Plant Soil.

[34] Parton W.J., Stewart J.W., Cole C.V. (1988). Dynamics of C, N, P and S in grassland soils: A model. Biogeochem..

[35] Zhen Q., Ma W., Li M., He H., Zhang X., Wang Y. (2017). Effects of vegetation and physicochemical properties on solute transport in reclaimed soil at an opencast coal mine site on the Loess Plateau, China. Catena..

[36] He Y., He X., Liu Z., Zhao S., Bao L., Li Q., Yan L. (2017). Coal mine subsidence has limited impact on plant assemblages in an arid and semi-arid region of northwestern China. Ecoscience.

[37] Zeng Q., Liu Y., Xiao L., Huang Y. (2017). How fencing affects the soil quality and plant biomass in the grassland of the loess plateau. Int. J. Environ. Res. Public Health.

[38] Li Y., Chen L., Wen H. (2015). Changes in the composition and diversity of bacterial communities 13 years after soil reclamation of abandoned mine land in eastern China. Ecol. Res..

[39] Pourhassan N., Bruno S., Jewell M.D., Shipley B., Roy S., Bellenger J.-P. (2016). Phosphorus and micronutrient dynamics during gymnosperm and angiosperm litters decomposition in temperate cold forest from Eastern Canada. Geoderma..

[40] Kadlec R.H. (2005). Phosphorus removal in emergent free surface wetlands. J. Environ. Sci. Heal. A.

[41] Liu X., Bai Z., Zhou W., Cao Y., Zhang G. (2017). Changes in soil properties in the soil profile after mining and reclamation in an opencast coal mine on the Loess Plateau, China. Ecol. Eng..

[42] Cheng J.X., Nie X.J., Liu C.H. (2014). Spatial variation of soil organic carbon in coal-mining subsidence areas. J. China Coal Soc..

[43] Wang P., Hu Z., Yost R.S., Shao F., Liu J., Li X. (2016). Assessment of chemical properties of reclaimed subsidence land by the integrated technology using Yellow River sediment in Jining, China. Environ. Earth Sci..

[44] Cambardella C.A. (1994). Field-scale variability of soil properties in central Iowa soils. Soil Sci. Soc. Am. J..

[45] Zhang Y.L., Chen L.J., Chen X.H., Tan M.L., Duan Z.H., Wu Z.J., Li X.J., Fan X.H. (2015). Response of soil enzyme activity to long-term restoration of desertified land. Catena.

[46] Kang Y.H., Liu S.H., Wan S.Q., Wang R.S. (2011). Assessment of soil enzyme activities of saline–sodic soil under drip irrigation in the Songnen plain. Paddy Water Environ..

[47] Stemmer M., Gerzabek M.H., Kandeler E. (1998). Organic matter and enzyme activity in particle-size fractions of soils obtained after low-energy sonication. Soil Biol. Biochem..

[48] García-Ruiz R., Ochoa V., Hinojosa M.B., Carreira J.A. (2008). Suitability of enzyme activities for the monitoring of soil quality improvement in organic agricultural systems. Soil Biol. Biochem..

